# ‘You try to play a role in her pregnancy’ - a qualitative study on recent fathers’ perspectives about childbearing and encounter with the maternal health system in Kigali, Rwanda

**DOI:** 10.3402/gha.v9.31482

**Published:** 2016-08-25

**Authors:** Jessica Påfs, Stephen Rulisa, Aimable Musafili, Birgitta Essén, Pauline Binder-Finnema

**Affiliations:** 1 Department of Women's and Children's Health/IMCH, Uppsala University, Uppsala, Sweden; 2 Department of Obstetrics & Gynecology, School of Medicine, College of Medicine and Health Sciences, University of Rwanda, Kigali, Rwanda; 3 Department of Clinical Research, University Teaching Hospital of Kigali, Kigali, Rwanda; 4 Department of Pediatrics and Child Health, School of Medicine, College of Medicine and Health Sciences, University of Rwanda, Butare, Rwanda

**Keywords:** male involvement, intimate relationship, masculinity, relational theory, gender, sub-Saharan Africa

## Abstract

**Background:**

Rwanda has raised gender equality on the political agenda and is, among other things, striving for involving men in reproductive health matters. With these structural changes taking place, traditional gender norms in this setting are challenged. Deeper understanding is needed of men's perceptions about their gendered roles in the maternal health system.

**Objective:**

To explore recent fathers’ perspectives about their roles during childbearing and maternal care-seeking within the context of Rwanda's political agenda for gender equality.

**Design:**

Semi-structured interviews were conducted with 32 men in Kigali, Rwanda, between March 2013 and April 2014. A framework of naturalistic inquiry guided the overall study design and analysis. In order to conceptualize male involvement and understand any gendered social mechanisms, the analysis is inspired by the central principles from relational gender theory.

**Results:**

The participants in this study appeared to disrupt traditional masculinities and presented ideals of an engaged and caring partner during pregnancy and maternal care-seeking. They wished to carry responsibilities beyond the traditional aspects of being the financial provider. They also demonstrated willingness to negotiate their involvement according to their partners’ wishes, external expectations, and perceived cultural norms. While the men perceived themselves as obliged to accompany their partner at first antenatal care (ANC) visit, they experienced several points of resistance from the maternal health system for becoming further engaged.

**Conclusions:**

These men perceived both maternal health system policy and care providers as resistant toward their increased engagement in childbearing. Importantly, perceiving themselves as estranged may consequently limit their engagement with the expectant partner. Our findings therefore recommend maternity care to be more responsive to male partners. Given the number of men already taking part in ANC, this is an opportunity to embrace men's presence and promote behavior in favor of women's health during pregnancy and childbirth – and may also function as a cornerstone in promoting gender-equitable attitudes.

## Introduction

Men's responsibilities in childbearing first appeared as a global policy agenda in 1994 at the International Conference on Populations and Development (ICPD) (1). Since then, numerous studies have demonstrated that men's involvement promotes positive health outcomes – not only for women, but also for men and their children (2–4). A father's involvement during pregnancy appears to contribute to women's uptake of maternal health services, in addition to increasing his understanding of potential risks and the necessity to seek timely care (5–7). Including men in pregnancy and caretaking activities challenges gendered roles and social structures and promotes gender-equitable attitudes, both of which pose interesting future directions (4, 8). However, there are settings where men report facing barriers to increased involvement. This is partly because of childbearing and its related phenomena being considered the female domain. Studies throughout sub-Saharan Africa, for example, identify how men are often bound to normative social and cultural contexts, fueled by gendered expectations, which are attitudes similarly upheld by health system regulations or health care staff attitudes (9–12).

Gender equality was recently added to the political agenda in Rwanda and is defined in the country's National Gender Policy document. This document states that ‘The issue of gender inequality is embedded in patriarchy as a system’ and identifies, among other things, a need to ensure that reproductive health services are accessible to both women and men (13). Another significant undertaking revisits inequitable laws and policies, where it was previously stated in the law that a husband had the dominating power in an intimate relationship; ‘The man is the head of the family and his opinion must prevail’ (14). However, this law is currently updated. Even though such structural changes are currently taking place, and more women are entering into paid work life, the promotion for gender equality has encountered some resistance within households and the community (15). A man is still viewed as the breadwinner, having dominance in decision-making, without expectation for becoming involved in caring activities (15, 16). Yet those Rwandan men having both higher education and income are identified as moving beyond traditionally gendered roles and espousing more gender-equitable attitudes (8, 17).

The health system in Rwanda has improved over the past years and decreased its maternal mortality ratio from 1,071 per 100,000 live births in the year 2000 to 210 in 2015 (18, 19). At present, up to 91% of women are giving birth in a health facility, which partly is explained by the rule that imposes a fine if women deliver at home, and the availability of health insurance (20). Currently, 73% of the population is covered by the community-based health insurance, ‘Mutuelle de Santé’ (herein, Mutuelles), covering 90% of the care costs and some medicines (21, 22). Rwanda also reports a distinctly high number of male attendants at first antenatal care (ANC) for HIV testing with up to 87% of women attending with their partner, which is distinctive for this sub-Saharan setting (23, 24). The policy of partner involvement had the intention to prevent mother-to-child transmission, but appears to have turned into a strict requirement for male attendance, a factor that might hinder women from attending alone (25). However, this may also, together with the new political agenda, prompt men to renegotiate definitions of masculinity and challenge traditional gender norms. This study therefore explores recent fathers’ perspectives about their roles during pregnancy, maternal care-seeking, and childbirth within the current context of Rwanda's political agenda for gender equality.

## Methods

### Conceptual framework

Because of the context of Rwanda with increased focus toward gender equality and involvement of men in ANC, we use Connell's relational theory of gender to conceptualize the participant's perspectives about their experiences (26, 27). This theory posits gender as socially constructed, and that men are gendered beings, having power as a central aspect of their relations, actions, and expectations. Gendered expectations are formed both under the influence and expectations of society, as well as by individual interactions, both between and among women and men. The additional concept of hegemonic masculinity embodies what is ‘currently accepted’ of a man in a certain context. Even though an individual's behavior is complex, and each individual will cope differently with the social setting, Connell argues that gendered structures produce a narrow arena for individuals. The construct of gender is thus multidimensional and arranged simultaneously on intrapersonal, interpersonal, institutional, and society-wide levels (26, 27). The pattern in gender structures on these different levels can be called gender regimes. Institutions, such as a health system, follow the logics of the gender regime within its specific context, and policies are simultaneously constructing and deconstructing the gendered order of a society.

### Ethical considerations

Ethics approval for the project was obtained from the Rwanda National Health Research Committee, Kigali (NHRC/2012/PROT/0045), as well as the Institutional Review Board of Kigali University Teaching Hospital. Permission to recruit participants was sought and obtained separately from each hospital. All participants were approached and included in this study following our precise adherence to the ethics approval, including informed consent for participation, and strict handling and storage of the recorded and transcribed interviews.

### Research design and sampling

This study took place in Kigali, the capital of Rwanda, having 1.2 million inhabitants and vast socioeconomic differences (28). The average inhabitant generates an income from farming or service-related industries, such as driving a motorbike or taxi. The literacy rate is 80% for men and 77% for women (19). Kigali provides public and private health centers, three district hospitals, and three tertiary referral hospitals.

Data were collected between March 2013 and April 2014 at three public hospitals in Kigali and this work is part of a project focusing on maternal near-miss (MNM) in the context of Rwanda. MNM refers to women who survive a severe, life-threatening obstetric complication during childbearing (29), and this approach is appropriate for identifying underlying factors to mortality, both including care-seeking delays and inadequate provision of care (30–32). Some participants were recruited purposively with the help of health care workers and others via snowball sampling within the community (33) with assistance from a Rwandan interpreter, fluent in both Kinyarwanda and English. The research team was composed of three female European researchers (a medical doctor, a medical anthropologist, and the doctoral student whose foundation is in social work) and two male medical doctors from Kigali (both holding a PhD in international health). The first author (the doctoral student) and a local interpreter conducted individual interviews with the partners of women who had experienced MNM while they were still at the hospital, yet only after the woman gave her consent for their inclusion. These men were also asked to bring one or two friends to join group interviews, which were conducted at a later session in the community, either at a participant's home or at a conference center. The inclusion criteria for all participants were having a partner who recently experienced MNM or having recently become a father. All except two men consented to take part after learning the aims of the study. No reasons were given for the two men who declined (Table 1).

**Table 1:** Overview of data collection

Participants	Data collected	*N* (type of data collection)	Length	Place
Partners to MNM	1–3 day(s) post-MNM	9 (IDI)	20–60 min	Hospital, private room
Partners to MNM	3–6 months post-MNM	4 (IDI, with MNM partner)	60–120 min	Participants’ home
Partners to MNM and snowballed participants	3–6 months post-MNM	3 (FGD; total 19 participants)	120–180 min	Conference center
Partners to MNM and snowballed participants	6–12 months post-MNM	3 (GD; total 8 participants)	90–120 min	Participants’ home

MNM=maternal near-miss; IDI=individual, semi-structured interview; GD=group discussion; FGD=focus group discussion.

The framework of naturalistic inquiry guided the study (34). A hermeneutic dialectical approach was used during the interviews for eliciting a conversation-like experience about how men perceived themselves in this shared context (34). All interviews were transcribed into English for analysis and cross-checked by an external translator, which was done to ensure the validity of the transcription (35). Themes of the semi-structured interview questions included men's perceived role during pregnancy, how they experienced their partner's pregnancy outcome – which was sometimes life-threatening, and their attitudes toward fitting in during the care encounter with medical professionals.

### Analysis

Naturalistic inquiry guided the emergent analysis (34), which began during initial data collection, where information from one interview helped to guide new questions in later interviews. Topical saturation of new concepts was met upon repetition of participants’ answers. We performed member checks to validate the findings (34), and the necessity for establishing topical saturation was met upon repetition of participants answers. In-depth analysis occurred once all interviews were transcribed via rereading of all interviews and use of AtlasTi (Scientific Software, 2013) for sorting the data. The first and last authors read the sorted data and coded and interpreted overarching categories that best encapsulated the men's perspectives about their role. These were then sorted into the conceptual framework inspired from Connell (26) and discussed by the full research team.

### Findings

**Fig. 1:**
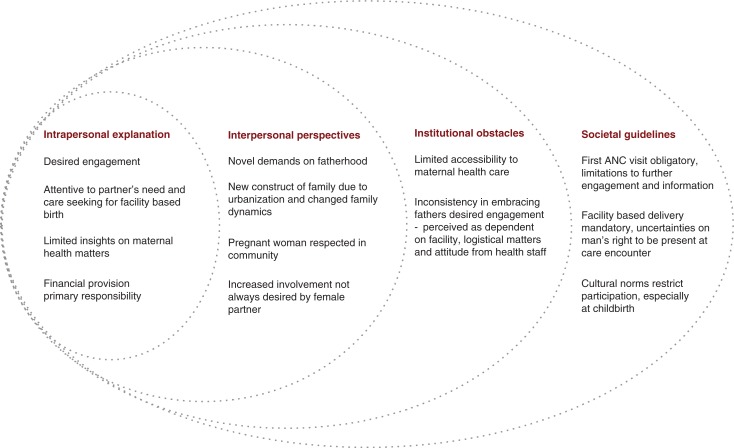
Conceptual framework, inspired from Connell (26), mapping recent fathers’ perceived roles during childbearing and maternal care-seeking, illustrated on the different levels.

Figure 1 details the outcome of data collection among 32 men, 13 of whom had a partner that experienced MNM. Four of these men's partners had had a miscarriage and three babies had not survived birth. Among the remaining participants, two had prior experiences of a baby not surviving birth. All of the men expressed their desire for the pregnancy, even if it was not necessarily intended. The men were between 23 and 35 years old, and two were in their 50s. One participant was currently studying at the university, whereas all the others had either primary or secondary level of education. The men had varied occupations, and several of them were either unemployed or were daily wageworkers. The men in this study were among those having the community health insurance Mutuelles, which is available for low-income households not covered by other insurance systems, such as those for employees of government, military, or private companies.

The men openly shared their reflections on their perceived role during their partners’ childbearing. However, the topic of the MNM caused a momentary quietness before the men expressed thoughts of anxiety and frustration over the near loss of their partner and the loss of their coming baby. The event seemed to have brought these men to reflect on their own participation and to be more attentive to flaws in the maternal health system. Men's perspectives were interpreted on an intrapersonal, interpersonal, institutional, and societal level in Fig. 1 and represented separately for clarity. However, these levels should be understood as interlinked.

### Intrapersonal explanations

A pregnancy was perceived as a blessing and embraced with joy, yet also as a responsibility embraced with worries. Men's main purpose was perceived as being the breadwinner, with the role to provide financially for their partner and coming family member. This role was not experienced as short term: ‘As a man you need to provide for the child until s/he is grown up enough to look after her/himself’ (28 years old, first child, partner to MNM). Nevertheless, it appeared important to be strong and engaged and to not allow anxieties about the new responsibility to affect the partner during pregnancy. When asked to define their role toward a pregnant partner, most men presented ideals of an engaged father, using attributes of caring and benevolence. Although some focused mostly on the financial aspects, several of the men further explained the importance of providing care so the woman could rest. Pressuring the partner for sex during pregnancy was seen as selfish, and referred to as something happening earlier, during traditional times, before men's understanding of the need to show respect toward a pregnant partner. The men were thoughtful about such attentiveness, but also toward consequences to the coming child:

[During pregnancy] you should listen to whatever your wife tells you, and stop thinking that accepting her ideas or the advice she gives you is some sort of unmanly attitude. You also have to avoid being rude towards her or frightening toward her, because if she gets angry it will affect the baby, too. And then you risk losing both of them because you were a jerk. (FGD 3)

Several men regarded their roles as dynamic, emphasized on shared decision-making, concluding that, ‘A man should stop thinking that he is the king of the home’ (FGD 2).

Participants felt constrained by their limited insights into maternal health matters, yet perceived themselves as responsible to ensure a woman received maternal care. This was particularly apparent for ANC because the men perceived their presence as mandatory during the first ANC visit. Attendance was understood as required for HIV testing, and ensuring the expectant partner would be received for consultation. The men described attendance at the first ANC as a ‘government rule’ requiring ‘obligatory involvement’. However, complaints were raised about how long wait times actually made men have to take 1 day free from work, which created a liability to their role as breadwinner. This was especially true among those who worked far from home, reflecting that their limited presence had delayed their partner's initial maternal care-seeking. The requirement for a man's attendance at the first visit was perceived as non-flexible at public clinics, which motivated some to ensure their partner went instead to a private clinic.

### Interpersonal perspectives

The men perceived new responsibilities during pregnancy. Their reflections about changed family dynamics were discussed through the lens of changing traditions. Many lived far away from family members after moving to Kigali to find employment, or they had few role models after having lost extended relatives in the 1994 Genocide. The men were particularly aware of the loss of their partner's mother or their own, that is, women who would have otherwise provided support for the pregnant partner. Men described their attempts to become involved, often consulting with relatives and friends to be able to assist: ‘You try to play a role in her pregnancy and you do all you can to make sure she is taken care of’ (33 years old, second child, partner to MNM). However, consulting with others was not always self-evident because pregnancy was esteemed a strictly private matter, shared between a man and woman. One man reflected, ‘Everybody manages their problems. You cannot tell the secrets of the family to other people’ (35 years old, second child, partner to MNM). At most, the couple could share with closest family or people considered trustworthy.

Obtaining information on maternal health was generally perceived as challenging and identified as posing potential difficulties on the intimate relationship. Only a few had been present when their partner received maternal health information from a care provider. Not being present at such times seemed to consequently prevent some from absorbing information, for example: ‘When she comes home and tells you what they have told her, you just listen [to her] but do not care, because you were not there’ (GD: 34 years old, third child). Although pregnancy was considered a private matter, a pregnant woman was seen as respected in the community, and men perceived themselves to be under external pressure to fulfill expectations about becoming a father. There was the need to be caring and attentive to a partner's needs, and making sure she attends ANC. A man not attending to these responsibilities risks being publicly referred to as either negligent or cowardly. Most men considered being involved and affectionate in parallel, saying, ‘If you cannot cope with your wife's troubles, then you do not love her’ (FGD 3).

### Institutional obstacles

Nearly all men perceived limited exposure to pregnancy information, as this was not provided at all facilities. They also had different experiences with ANC. Only a few had been included in a group information session conducted at the care facility, covering pregnancy and health risks. None of the men were welcomed during the actual pregnancy consultation, wondering aloud why they could not participate and receive direct, first-hand information from the care provider. For example, ‘[Health care workers] are more interested in talking to the women, but they do not consider informing the men, as well. It would be better if we all could understand more about those symptoms’ (GD: 27 years old, second child).

Participants were strongly in favor of facility-based childbirth. They viewed it as their responsibility to bring a woman to a health facility where she would be taken care of by professionals, but also wished to avoid the imposed fine if she delivered at home. The latter was perceived as a bother that required strategizing:

You have to keep the umbilical cord uncut and just rush to the hospital, because if you cut it, you will have some issues with the doctors. They are going to fine you. You just need to explain to them that the contractions were sudden and took her by surprise. (GD: 28 years old, first child)

Several had accompanied their partner to the health facility at time of delivery, whereas others gave the responsibility to a female relative. Among the men who had accompanied their partners, some emphasized the importance of being there because ‘as a man, you will be listened to’ (FGD2). Many expressed bottlenecks at admission, doubt in the quality of maternal care provided and questioned care provider attitudes. For example, ‘[My wife] told me that when a woman is still in the waiting room and starts shouting, the [health care workers] will not even glance at her. They say that, “if you can still shout, it means you definitely still have strength”’ (33 years old, third child, one died during birth, partner to MNM). Another said his wife had been left alone in the ward and she had given birth while unattended. He explained, ‘I think that the medical staff should be trained in giving better care to their patients’ (GD: 31 years old, third child). One man contemplated that he, as the partner and man, might have been listened to by the care provider, in the case where his partner felt uncomfortable about expressing her needs: ‘There are some women who do not like to scream when they are in pain, which the doctor will interpret as if she is not suffering. But if you, who is closest to her, are there, then she can tell you to advise the doctor’ (26 years old, second child, first died, partner to MNM).

The idea of not being heard during the care encounter triggered a lack of trust and a wish to be present during labor. Yet, such presence at a public health care facility was perceived as non-negotiable and only allowed at private facilities. Some had not even questioned being barred from attendance during the birth, perceiving it as ‘not allowing men in is a law at the hospital, and so we have just gotten used to it’ (GD: 31 years old, third child). Other men reasoned their exclusion for logistical causes and the limited privacy found in the labor ward: ‘[Health care workers] do not want to violate the privacy of other women in the shared room’ (FGD 2). Even though the men were unable to surveil the actual procedure, they were held to a sense of responsibility. In one case, where a man was asked to sign a consent form for cesarean section, he raised this paradox:

They ask you to give your ID number and sign that you are there to make sure she is safe. But, then, after you sign, they turn around and lock you out of the delivery room. So, how am I supposed to ensure that my wife and kid are safe if am locked out of that room? (29 years old, first child, partner to MNM)

### Societal/cultural guidelines

Despite having limited accessibility on the institutional level, the participants knew of supportive policy guidelines, particularly regarding men's presence for the first ANC visit. Yet, a man's actual *right* for being present at the consultation and childbirth remained unclear and seemed undefined. This presented strong reasons for why men were hesitant to demand increased involvement as it also clashed with cultural norms, as one man explained:

In our culture, they say that if the husband sees his wife giving birth, he is never going feel attracted to her again sexually. We are saying that we should be there, but in the Rwandan culture, it is taboo to see your wife giving birth. Some women would not even accept to have their husbands there. So we are kind of on the fence. (26 years old, second child, first died, partner to MNM)

Several participants discussed the cultural aspects of women not wanting the partner to be present during consultation. More than simple tradition of preferring female attendants, in particular, men perceived women as not wanting their partners to witness them in the condition of giving birth. Most men also reflected and were in support of the idea that their presence should always be based on the consent of the expectant mother. A father of three children expressed a clear wish to participate, yet highlighted: ‘I think the decision should come from her. If she is comfortable with it, then [the health care workers] should accept her choice to allow whoever she came with to be there and to watch the entire procedure’ (GD: 34 years old). The men's clear motivation was a wish to surveil the birth procedure, especially among the men whose baby had not survived. One explained, ‘I should have been allowed in there so that I could be sure that whatever happened was nobody's fault’ (33 years old, third child, one died during birth, partner to MNM). A number of men from the public facilities reflected about not having the same access to the childbirth as was possible at private care facilities. They highlighted a wish to be financially able to seek private care. Moreover, men expressed a wish to challenge restrictions at public care facilities, but felt limited in their ability to negotiate and instead blamed the unclear execution of guidelines at these institutions.

## Discussion

These recent fathers in Kigali expressed support for increased engagement during pregnancy and childbirth, as also reported from other African settings (9, 11, 12). The participants voice sheds light on the perspective among men from the lower socioeconomic group in Rwanda and can be interpreted to contradict earlier findings arguing that men of lower education presented more conservative ideas (17). The participants described new family dynamics, which appeared to place novel demands on fatherhood. This also seemed to pave the way for changing role dynamics confronting their perceived rationale for a more traditional gender regime at the institutional and societal levels. However, these men appeared to balance their participation according to their perceived outer demands and cultural norms. The institutions appeared to vary in how willing they were to allow the men to take part, providing an inconsistent, and narrow, arena for increased engagement. This seemed to pose barriers for men as they attempted to access information about pregnancy health and risks.

The men's expressed rhetoric in favor of being caring and benevolent, and their reflections about moving beyond unmanly attitudes or being ‘the king of the home’, suggests an increasingly acceptable attitude that challenges traditional masculine ideals (36). Our findings suggest that changed family dynamics, particularly those due to the genocide, which caused disruption in the generational shifts, as well as migration from the rural to the urban setting, have partly changed the requirement for men to take a more active role during pregnancy. Another important aspect appears to be the current societal demand for participation at first ANC visit for HIV testing. As supported by other studies (37), this seems to have challenged earlier ideals of masculinity as well as participation as something socially expected from an expectant man. However, at present, a man's decision-making power is upheld because a woman in this setting will not be admitted if she seeks ANC alone (25). Arguably, the men's increased engagement may be grounded in a latent interest to retain power over women (4, 38). However, it is notable that the participants in our study maintained a concern for the welfare of their partners and seemed to challenge the social expectations placed on them. And where some of the men seemingly dismissed their partners’ needs after not receiving first-hand information, this may be interpreted as distrust with the intimate partner or a need to possess power. This aspect points out the challenges of gendered influences on intimate communication between the couple, which is essential during childbearing (16).

The men in this study appeared to have faced institutional barriers, despite their expressed wishes for increased involvement. There is, to our awareness, no written policy prohibiting men from being present during a woman's consultation and childbirth. It is thus presently up to the health facility and its staff to decide, which identifies a potential system-level inconsistency between institutions. The participants’ perspectives about health provider's unwillingness to include and engage them may reflect health providers’ own ‘traditional’ ideals of gendered masculinity. Alternatively, men might be unwelcomed in order for health care staff to protect their professional domain or to uphold maternity as a female domain. Most health care staff in Rwanda's maternity wards are women. If they are unwilling to renegotiate gender structures, this could also be a wish to uphold the power they currently possess over the patient. Moreover, having men present on the ward could be perceived as threatening to that established power and is a subject for further research. However, from the men's perspectives, their feelings of not being welcomed could have prevented them from challenging the established system. This is a subject for further research.

Our participants raised their concerns about lack of respect given to their partners during the care encounter, echoing current advice that emphasizes increased focus on offering respectful, woman-centered maternal care (39). The men's wishes for increased presence and surveillance could be triggered by their distrust in care quality, particularly among those having a partner facing severe morbidity or mortality. The institutional demand placed on the man to sign a document admitting he is responsible for the outcome places him in a paradoxical position. Nevertheless, an important aspect of the maternity care wards in Rwanda's public health facilities is that they have limited physical space to allow men in without risking the privacy of other women. This well-known reason for restricting men cannot be neglected (40). However, asking him to sign a document of responsibility might be received as being placed into a powerless position. These aspects highlight the men's desire to afford private care because these venues are perceived as providing better quality care, as well as allowing them to be present during childbirth (41). This socioeconomic disadvantage could consequently underpin the masculine ideal of being the financial provider.

Despite the push for gender equality, the lack of explicitly stated public policy about men's involvement at health care facilities in Rwanda contributes to men's disconnection and limits their access to pregnancy information. The men perceived these barriers as being regulated from the maternal health system, which is a situation likely to uphold current gendered domains. Notably, women may not be entirely supportive of challenging the gendered domains, either. Doyle et al. (16) identified Rwandan women as opposing increased male involvement during child care, because they did not want to be reflected upon poorly within their community. It is most likely that women are responding to the gendered expectations placed on them by society and tradition, even though this means they may unwittingly support gender inequitable norms. It is therefore essential to be attentive to the gendered ‘ideal of femininity’ in a setting. The maternal domain, specifically, may be one of the few arenas where a woman feels in power (37). Hence, male involvement should not compromise women's autonomy and decision-making. The men in this study have nevertheless shown themselves to be concerned about pregnancy, as well as the overall health of their partner and child during pregnancy, while also maintaining that women should have autonomous power to choose who is present during consultations and childbirth. However, with the narrow arena currently provided by the health system, it might bring such consequences as decreased motivation for involvement or could trigger distrust in both the available care and partner.

### Methodological considerations

Topical saturation was quickly reached, suggesting strong similarities of perspectives among these men within this peri-urban setting, regardless of whether their partner had a near-miss or not. However, those men partnered with women experiencing complications presented more negative ideas of the health system, which should be considered. These men's experiences about the near-miss event itself are presented only briefly because this is analyzed more in-depth in a separate paper. Face-to-face interviews may provide normative responses of social desirability; however, this in turn tends to mirror the hegemonic norm within a study context. These findings should be considered for this aspect. The design allowed several participants to be revisited, which lent opportunity to respond to earlier reflections and gain trustworthiness of the interpretations. Our study encountered a few limitations because of its reliance on language interpretation. However, the Rwandan co-authors assisted in securing experienced interpreters, and multiple Kinyarwanda-English transcripts were randomly cross-checked for validity using different translators, as recommended for interpreter use (35). The first author and data collection translator are women, which may have impacted the way male participants responded. However, this aspect was not experienced as a barrier for in-depth discussion. Additionally, the foreign nationality of the first author seemed to make men eager to explain their perspectives and experiences to her as an ‘outsider’. Yet, it might be that the first author's cultural background influenced her choice of probing follow-up questions. Thus, to help ensure trustworthiness, the interpretations were member-checked throughout the study, both among the participants and within the community. Importantly, the author also spent a total of 1 year in the setting and took the opportunity to member-check the researchers’ interpretations throughout her time in Rwanda. Another potential limitation is that men's precise caretaking activities are not included here because of being beyond the study's scope. This aspect could have enriched attitudes related to gender equality in this setting. Moreover, a deeper exploration on women's and health care providers’ perspectives are to be explored further in a separate study.

## Conclusion

Our findings imply that these men see an engaged and caring partner as the ideal, yet are facing challenges due to normative gendered expectations in this Rwandan society. From the viewpoint of our participants, they face resistance from the current maternal health policy and care providers in their desire to become increasingly involved. There are hints that the men's limited access and insight may motivate their mistrust of the public health system and its health care staff, but also of their intimate partner. This calls for attention in building men's trust in public maternity care as well as to the quality of care provided to help ensure safety for the pregnant woman. Additionally, because the maternal health system is a core social institution, it plays an important role in challenging gendered structures. The engagement of fathers during childbearing and maternal care-seeking may function as a cornerstone in promoting gender-equitable attitudes. With a number of men already attending ANC in this setting, the opportunity is already in place to inform and motivate men about their role as partner and to promote behaviors that favor women's health during pregnancy and childbirth. Men's interest for increased involvement is evident from our findings, which suggests their willingness to be responsive in a gender sensitive manner to women's needs in maternity care. However, policies aimed at increasing male involvement need to take into account that the inclusion of men could come at the cost of women's empowerment, particularly if a woman is not allowed to seek maternity care alone or with the person of her preference.

## Paper context

Male involvement can promote positive maternal health outcomes. With the increased focus on gender equality in Rwanda, this paper present men’s reflections on their roles during childbearing. While an engaged and caring partner was presented as the ideal, gendered expectations from the maternal health system and society were perceived as limiting this ideal. To promote gender equitable ideas, maternity care could be more responsive for men to actualize their wish for increased engagement.
